# Psychological Impact of Coronavirus Disease 2019 Among Italians During the First Week of Lockdown

**DOI:** 10.3389/fpsyt.2020.576597

**Published:** 2020-09-30

**Authors:** Mariagrazia Di Giuseppe, Sigal Zilcha-Mano, Tracy A. Prout, John Christopher Perry, Graziella Orrù, Ciro Conversano

**Affiliations:** ^1^ Department of Surgical, Medical and Molecular Pathology, Critical and Care Medicine, University of Pisa, Pisa, Italy; ^2^ Department of Psychology, University of Haifa, Haifa, Israel; ^3^ Ferkauf Graduate School of Psychology, Yeshiva University, Bronx, NY, United States; ^4^ Institute of Community and Family Psychiatry, JGH, McGill University, Montreal, QC, Canada

**Keywords:** coronavirus disease 2019, post-traumatic stress, psychological distress, emotion regulation, defense mechanisms, quarantine, pandemic (COVID-19), lockdown

## Abstract

Pandemics and government-mandated quarantining measures have a substantial impact on mental health. This study investigated the psychological impact of the coronavirus disease 2019 (COVID-19) crisis on Italian residents during the first week of government-imposed lockdown and the role of defense mechanisms as protective factors against distress. In this cross-sectional study, 5,683 Italians responded to an online survey assessing socio-demographics, overall psychological distress, post-traumatic symptoms, and defense mechanisms using validated measures as the *Symptom Checklist-90* (SCL-90), the *Impact of Event Scale-Revised* (IES-R), and the *Defense Mechanisms Rating Scale-Self-Report-30* (DMRS-SR-30). Data were collected from March 13 to March 18, within the first week of lockdown in Italy. Results showed that younger age and female gender were associated with increased psychological distress. Having positive cases nearby, more days on lockdown, and having to relocate were also associated with greater distress. Higher overall defensive functioning (ODF) was associated with lower levels of depression (*r* = −.44, 95% CI −0.48, −0.40), anxiety (*r* = −.38, 95% CI −0.42, −0.35), and post-traumatic stress symptoms (PTSS) (*r* = −.34, 95% CI −0.38, −0.30). Conversely, less adaptive defensive functioning was related to greater affective distress across all domains. Each increased unit of ODF decreased the chances of developing post-traumatic stress symptoms (PTSS) by 71% (odds ratio = 0.29, *p* < 0.001, 95% CI.026,.032). The psychological impact of COVID-19 among Italians during the early weeks of government lockdown has been significant. The pandemic continues to have extraordinary mental health impact as it moves across the globe. Given the salience of defensive functioning in psychological distress, consideration of interventions that foster the use of more adaptive defenses may be an important component of building resilience amidst a pandemic.

## Introduction

In March 2020, the World Health Organization declared coronavirus disease 2019 (COVID-19), the disease caused by severe acute respiratory syndrome coronavirus 2 (SARS-CoV-2), a pandemic. At the time of conducting this study only few countries registered positive cases and deaths for COVID-19, with Italy being the first hit European country (1). Disasters, like the one currently unfolding across the world, adversely affect well-being and overall mental health, requiring an immediate international response from multidisciplinary mental health science (2–4). The rapid spread and devastating impact of COVID-19 has resulted in social distancing, self-quarantining, and government-enforced lockdown of citizen movement. On March 9, 2020, the Italian government expanded local lockdown efforts to include all localities, requiring more than 60 million people to stay at home. The aim of this study was to assess psychological effects associated with the pandemic and their relationship to demographic and COVID-19 impact variables.

Many studies have highlighted the negative psychological impact of quarantining in concert with the obvious public health benefits (5, 6). Quarantining, coupled with other risk factors, during the severe acute respiratory syndrome (SARS) epidemic increased the odds of depressive symptoms 3 years later and a 2 to 3-fold increase in post-traumatic stress symptoms (PTSS) (7–9). Social isolation, stress, and anxiety were also associated with higher suicide rates among survivors of the SARS outbreak (10, 11). Proximity to intense outbreaks of an epidemic was also associated with higher rates of anxiety (12).

Individuals experiencing stressful life events rely on a wide range of explicit and implicit coping strategies. Active coping strategies have been shown to buffer against the impact of living amidst a global epidemic (13). Conversely, avoidant coping strategies, those that help individuals reduce emotional stress rather than dealing directly with a stressful situation, are associated with poorer psychological outcomes (13–17). Individuals who engaged in altruistic acceptance of risk during the SARS epidemic experienced lower levels of PTSS (9). The stressors associated with social isolation, quarantining, and exposure to a global health crisis do not end when the pandemic is over. Among survivors who were directly affected by the SARS epidemic, 17% had not returned to work after 1 year and had significant reductions in mental health that persisted 1 year later (18). These findings point to the importance of understanding the automatic coping mechanisms that people may employ under intense stress provoked by pandemic.

Implicit emotion regulation capacities are a central and salient aspect of how individuals manage traumatic events (19–21), particularly because they are employed automatically and often unconsciously. These cognitive and affective processes are vital components of self-regulation and mental health (22–26). There is evidence suggesting that implicit emotion regulation may be even more important to healthy mental functioning than explicit emotion regulation mechanisms (27). The inherent links between implicit emotion regulation and defense mechanisms have been explicated in detail (28). Assessing psychiatric distress and automatic mechanisms of emotion regulation, such as defense mechanisms, during the current global pandemic is of critical importance.

### Objectives

Among Italians responding during the COVID-19 outbreak, this study sought to 1) examine the prevalence of symptoms of psychological distress and identify predictors of distress; and 2) evaluate different associated emotion regulation strategies (operationalized as defense mechanisms) that might impact the relationship between stress and distress. Specifically, we asked whether specific defenses might moderate the association between knowing individuals positive for COVID-19 and greater levels of symptoms of post-traumatic stress disorder (PTSD) and distress.

## Methods

### Participants

Participant characteristics are presented in **Table 1**. Respondents (*N* = 5,683) were living in Italy, mostly young and middle-aged adults, female, living with close relatives, and without children. Their provenience was northern Italy (23%), central Italy (55%), and southern Italy (22%). Only 4% moved to another city as a result of COVID-19 and 62% were able to work remotely. Because data were collected during the first week after the Italian government decreed highly restrictive norms for all inhabitants, only 34% of participants reported being on lockdown for 2 weeks or more. A small percentage of participants endorsed being diagnosed with COVID-19 (6%, *N* = 329) or experiencing the death of close relatives or friends due to the virus (2%, *N* = 108).

**Table 1:** Demographics and coronavirus disease 2019 (COVID-19)-related characteristics (N = 5,683).

Variable	N	%
Age		
<30	1,935	34
30–39	1,179	21
40–49	1,032	18
50–59	954	17
>=60	582	10
Gender		
Male	1,427	25
Female	4,256	75
People living at home		
Close relatives	4,023	71
Partner/lover	746	13
Roommates	254	4
Alone	660	12
Positive cases among relatives and friends		
No	5,354	94
Yes	329	6
Days on lockdown		
<=7 days	3,772	66
8–14 days	1,568	28
> 14 days	343	6
Relocated		
No	5,450	96
Yes	233	4
Death among relatives and friends		
No	5,575	98
Yes	108	2
Working remotely		
No	2,137	38
Yes	3,546	62
Children		
No	3,387	60
Yes	2,296	40

### Measures

Participants provided socio-demographic information, the presence/absence of positive cases or deaths among relatives and friends, and whether they had moved to another location due to COVID-19. Psychological distress, post-traumatic symptoms, and implicit emotion regulation were assessed using Italian validated version of the Symptom Checklist-90, the Impact of Event Scale-Revised, and the Defense Mechanisms Rating Scales-Self-Report-30, respectively.

The *Symptom Checklist-90* (SCL-90) (29) is a 90-item 5-point scale assessing psychopathological and somatic symptoms occurring during the past week. The SCL-90 provides a Global Severity Index (GSI) and nine subscale scores for psychiatric symptoms, such as somatization, obsessive compulsive disorder, interpersonal sensitivity, depression, anxiety, hostility, phobic anxiety, paranoid ideation, and psychoticism. We focused on the subscales for depression (DEP) and anxiety (ANX) as well as the summary Global Severity Index (GSI). Validity and reliability of the scale are well-documented (30, 31).

The *Impact of Event Scale-Revised* (IES-R) (32) is a 22-item scale assessing the presence of post-traumatic symptoms. The IES-R provides an overall index of PTSS with three subscales reflecting intrusion, avoidance, and hyperarousal. The IES-R has performed well as a screening instrument for PTSD, and has demonstrated concurrent and discriminant validity, as well as a lack of social desirability effects (33).

The *Defense Mechanisms Rating Scales*-*Self-Report-30* (DMRS-SR-30) (34) is a 30-item questionnaire assessing the whole hierarchy of defense mechanisms (35). The DMRS-SR-30 items were extracted from the observer-rated Defense Mechanisms Rating Scales Q-sort version (DMRS-Q) (36) for use in self-report. The DMRS-SR-30 provides scores for the overall defensive functioning (ODF) and for seven hierarchically ordered defense levels. The defense levels (and constituent defenses), from least to most adaptive are: action (acting out, passive aggression, and help-rejecting complaining), major image-distorting (splitting of self and others’ images, and projective identification), disavowal (denial, rationalization, projection, and autistic fantasy), major image-distorting (idealization of self and others’ images, devaluation of self and others’ images, and omnipotence), neurotic (repression, dissociation, reaction formation, and displacement), obsessive (undoing, intellectualization, and isolation of affects), high-adaptive (affiliation, altruism, anticipation, humor, self-assertion, self-observation, sublimation, and suppression). Preliminary analysis of reliability showed very good internal consistency for ODF (Cronbach’s alpha = .890), whereas defense level subscales ranged from .360 to .703 (34). Similarly, very good criterion, concurrent, convergent and discriminant validity for ODF and moderate to high for defense levels subscales (34).

### Procedures

This cross-sectional study used snowball sampling *via* social media (e.g., Facebook, Instagram, Twitter) within Italy for data collection. An online survey about the psychological impact of quarantine for COVID-19 outbreak was launched on March 13, 2020 and data was collected for five days, during the first week of the Italian government lockdown decree. Considering the difficulty in enrolling participants under such restrictive measures, we opted for an Internet-based snowball sampling to collect self-report of how the COVID-19 affected participants. The high rate of response (0.0001% of Italian permanent residents of all ages) and the sample stratification by age and region provided an overall picture of the early psychological reaction to the COVID-19 pandemic and associated quarantining. Eligibility criteria for participation was: 1) consent to data being used for research purposes; 2) 18 years or older; and 3) living in Italy during the COVID-19 lockdown. All procedures followed the ethical standards of the Helsinki Declaration and were approved by the institutional review board at University of Pisa.

### Statistical Analyses

Stepwise linear regression was used to predict mental health symptoms (GSI and IES) by COVID-19 exposure and demographic variables. Exposure to COVID-19 was calculated as a percentage, with the daily incidence of positive cases in each of 20 Italian regions divided by the total of confirmed COVID-19 cases in Italy, on the day the participant completed the survey (data extracted from Italian Ministry of Health website: https://bit.ly/3dB7t3r). To predict PTSS by COVID-19 exposure, demographics, and overall defense functioning (ODF), stepwise logistic regression was used. Participants were also asked, “Do you have any of your relatives or friends who tested positive to COVID-19?” Responses to this question were classified as “having positive cases nearby” and it was treated as a categorial variable. The stepwise selection method had variable entering criteria of 15% and stay criteria of 5% significance level of the Wald chi-square. To evaluate the impact of defense mechanisms (i.e., implicit emotion regulation), ODF and DMRS-SR-30 subscales were tested as moderators using multivariate linear regression.

## Results

Exposure to COVID-19 among participants was 6.4% (*SD* = 11.7%) on average. Psychological distress was, on average, within the normal range, with GSI and IES-R mean scores (*M* = 0.72, *SD* = 0.53 and M = 24.72, *SD* = 16.10, respectively) below the cut-off for psychopathology. Within this sample, 35.6% reported clinically significant levels of distress, measured by the GSI, 29.4% reported clinically significant symptoms of post-traumatic stress, as measured by the IES-R. According to literature, cutoff scores for clinical significance were GSI > 0.8, IES-R > 33, DEP > 1.04, and ANX > 0.68 (37–39). Significant symptoms of depression and anxiety were reported by 37.8 and 51.1% of participants respectively. Overall defensive functioning fell in the healthy-neurotic range, comparable to a normative community sample (*M* = 5.7; *SD* = 0.70) (40). Participants with IES-R scores falling in the PTSD range showed significantly lower ODF (*M* = 5.21; *SD* = 0.62; *p* = .000) and GSI (*M* = 1.24; *SD* = 0.53; *p* = .000).

Results of linear regression are displayed in **Table 2** and indicated that COVID-19 exposure was not a significant predictor of symptoms. However, having positive cases nearby, more days on lockdown, and having to move because of COVID-19 were related to higher symptoms. Older age, working from home, male gender, and not living with close relatives were related to lower symptoms. Findings remained the same when treating age as a continuous variable.

**Table 2:** Linear regression for demographic variables predicting distress (N = 5,682).

	Psychological distress (GSI)		PTSD symptoms (IES-R)	
	*SE*	β	*SE*	β
Age				
< 30				
30–39	0.021	−0.094^***^	0.595	−0.960
40–49	0.024	−0.133^***^	0.613	−1.144^*^
50–59	0.026	−0.216^***^	0.631	−3.396^***^
>= 60	0.030	−0.302^***^	0.751	−7.005^***^
Female	0.016	0.185^***^	0.477	8.379^***^
Living with				
Close relatives				
Alone	0.023	−0.022	0.660	−1.394^**^
Partner	0.022	−0.088^***^	0.635	−1.481^**^
Roommates	0.034	0.047	1.020	−0.011
Close positive cases			0.883	2.165^**^
Lockdown duration				
<=7 days				
8–14 days	0.015	0.052^***^	0.470	1.299^***^
>14 days	0.029	0.139^***^	0.878	2.775^***^
Moved to new location	0.034	0.090^***^		
Working remotely	0.014	−0.045^***^	0.438	−1.233^***^
Children	0.020	−0.051^**^		
Constant	0.020	0.723^***^	0.617	20.616^***^
R^2^		0.084		0.080
Adjusted R^2^		0.082		0.078

*p < 0.05 ^**^p < 0.01 ^***^p < 0.001, two-tailed.

**Table 3** shows findings concerning the second hypothesis suggest that the likelihood of developing PTSD increased significantly for ages 30–39 (*OR* = 1.22) and 40–49 (*OR* = 1.35) compared to age <30, and decreases by 48% for age > 60, compared to age <30. Females (*OR* = 2.72) and participants who have positive cases nearby (*OR* = 1.44) show higher likelihood of PTSD. Higher ODF values are related to decreased likelihood of PTSD. Each increase of one unit of ODF results in a decreased the chance of developing PTSD in 71% (*OR* = 0.29, *p* < 0.001).

**Table 3:** Comparison of parameters and association with post-traumatic stress disorder (PTSD) symptoms.

	No PTSD (IES-R<33) (*N* = 4,012)	PTSD (IES-R >= 33) (*N* = 1,671)	*p*
Age			<0.001
<30	1,280 (31.9%)	655 (39.2%)	
30–39	808 (20.1%)	371 (22.2%)	
40–49	712 (17.8%)	320 (19.2%)	
50–59	707 (17.6%)	247 (14.8%)	
>=60	504 (12.6%)	78 (4.67%)	
Gender			<0.001
Female	2,803 (69.9%)	1,453 (87.0%)	
Male	1,209 (30.1%)	218 (13.0%)	
Relocated			0.013
No	3,865 (96.3%)	1,585 (94.9%)	
Yes	147 (3.66%)	86 (5.15%)	
Living with			0.033
Alone	497 (12.4%)	163 (9.75%)	
Close relatives	2,826 (70.4%)	1,197 (71.6%)	
Partner/lover	516 (12.9%)	230 (13.8%)	
Roommates	173 (4.31%)	81 (4.85%)	
Close positive cases			0.010
No	3,801 (94.7%)	1,553 (92.9%)	
Yes	211 (5.26%)	118 (7.06%)	
Close death			0.038
No	3,946 (98.4%)	1,629 (97.5%)	
Yes	66 (1.65%)	42 (2.51%)	
Lockdown days			0.008
<=7	2,702 (67.3%)	1,070 (64.0%)	
8–14	1,090 (27.2%)	478 (28.6%)	
> 14	220 (5.48%)	123 (7.36%)	
Working remotely			0.667
No	1,501 (37.4%)	636 (38.1%)	
Yes	2,511 (62.6%)	1,035 (61.9%)	
Children			<0.001
No	2,315 (57.7%)	1,072 (64.2%)	
Yes	1,697 (42.3%)	599 (35.8%)	
GSI	0.50 (0.35)	1.24 (0.53)	<0.001
ODF	5.77 (0.71)	5.22 (0.61)	<0.001
COVID-19_impact	6.51 (12.0)	6.10 (10.8)	0.212

**Table 4** displays the correlations between the defense variables and both overall psychological distress (GSI), depression (DEP), anxiety (ANX), and PTSD symptoms (IES-R). Overall defensive functioning (ODF) was significantly negatively related to symptom levels on all measures, in particular with overall distress and depression. Conversely, all lower/immature defense levels were positively related to symptom levels. Of the defense categories, mature defenses were negatively correlated with symptom scales, whereas neurotic and immature categories were positively associated with psychological distress and symptoms. In descending order of magnitude, they were: depression, anxiety, and PTSD symptoms. The immature defense category displayed the largest positive association to symptoms levels, of which the subgroup of depressive defenses showed the higher correlation compared to non-depressive defenses.

**Table 4:** Pearson correlations between psychological distress, post-traumatic stress disorder (PTSD), and defense mechanisms.

	Overall distress (GSI)	Depression (DEP SCL-90)	Anxiety (ANX SCL-90)	PTSD (IES-R)
ODF	−.506^*^	−.441^*^	−.381^*^	−.341^*^
Defense levels				
High-adaptive	−.563^*^	−.490^*^	−.431^*^	−412^*^
Obsessional	.151^*^	.138^*^	.118^*^	.129^*^
Neurotic	.320^*^	.281^*^	.262^*^	.290^*^
Hysterical	.335^*^	.286^*^	.260^*^	.260^*^
Other neurotic	.151^*^	.138^*^	.135^*^	.135^*^
Minor I-D	.206^*^	.156^*^	.135^*^	.134^*^
Disavowal	.306^*^	.270^*^	.253^*^	.266^*^
Major I-D	.549^*^	.498^*^	.419^*^	.348^*^
Action	.369^*^	.320^*^	.279^*^	.238^*^
Defense categories				
Mature	−.563^*^	−.490^*^	−.431^*^	−431^*^
Neurotic	.309^*^	.275^*^	.249^*^	.275^*^
Immature	.569^*^	.494^*^	.434^*^	.399^*^
Depressive	.597^*^	.531^*^	.449^*^	.377^*^
Other immature	.208^*^	.161^*^	.167^*^	.205^*^

*p < .001; image distortion abbreviated I-D.

**Figure 1:**
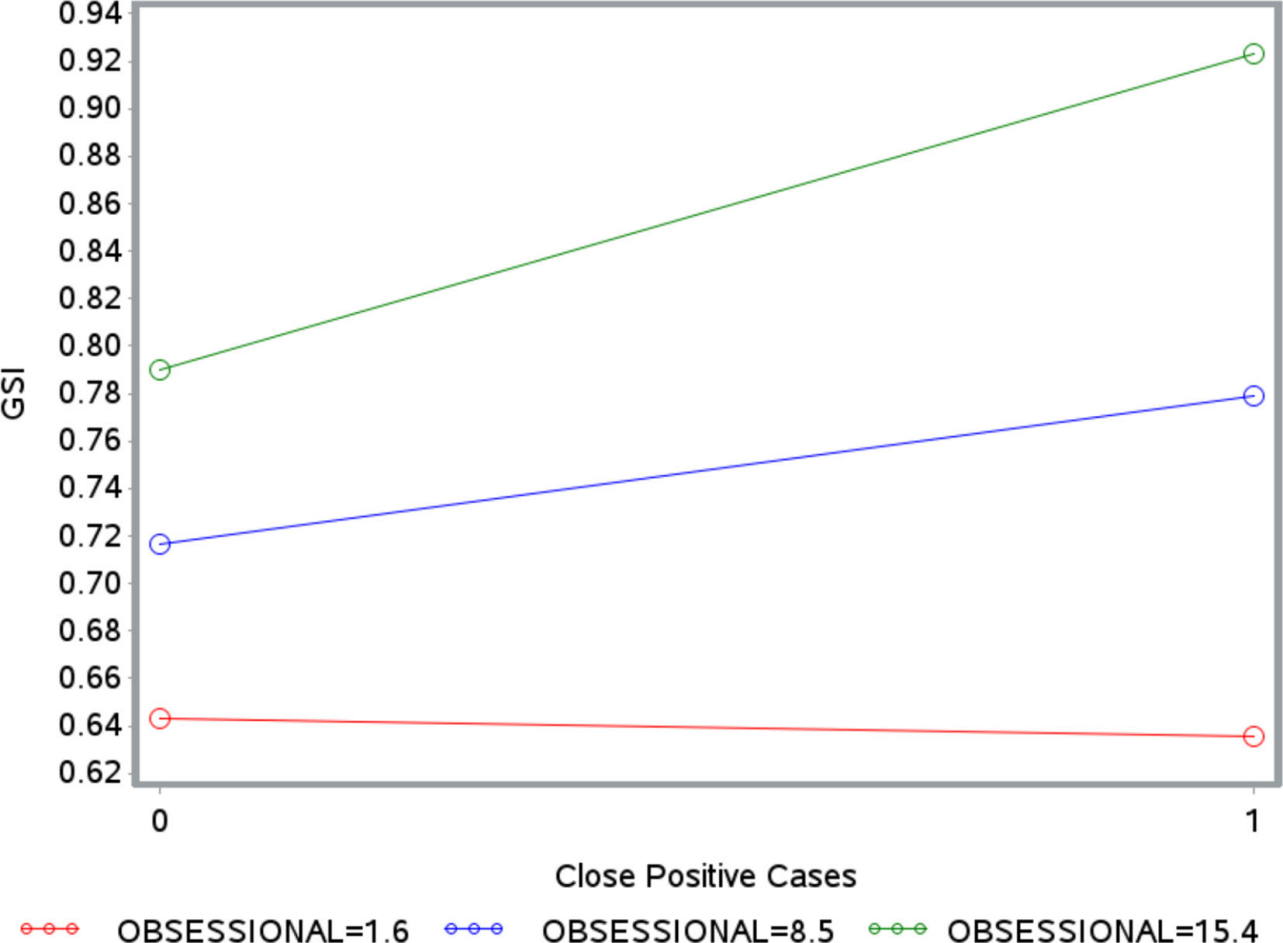
Moderating effect of obsessional defenses on the association between close positive cases and Global Severity Index (GSI). The number 0 on the x-axis indicates the absence of close positive cases. The number 1 on the x-axis indicates the presence of close positive cases. The label OBSESSIONAL refers to defense mechanisms belonging to the obsessional defense level. The red line, blue line, and green line indicate values of obsessional defense level of 1.6, 8.06, and 15.4%, respectively.

**Figure 2:**
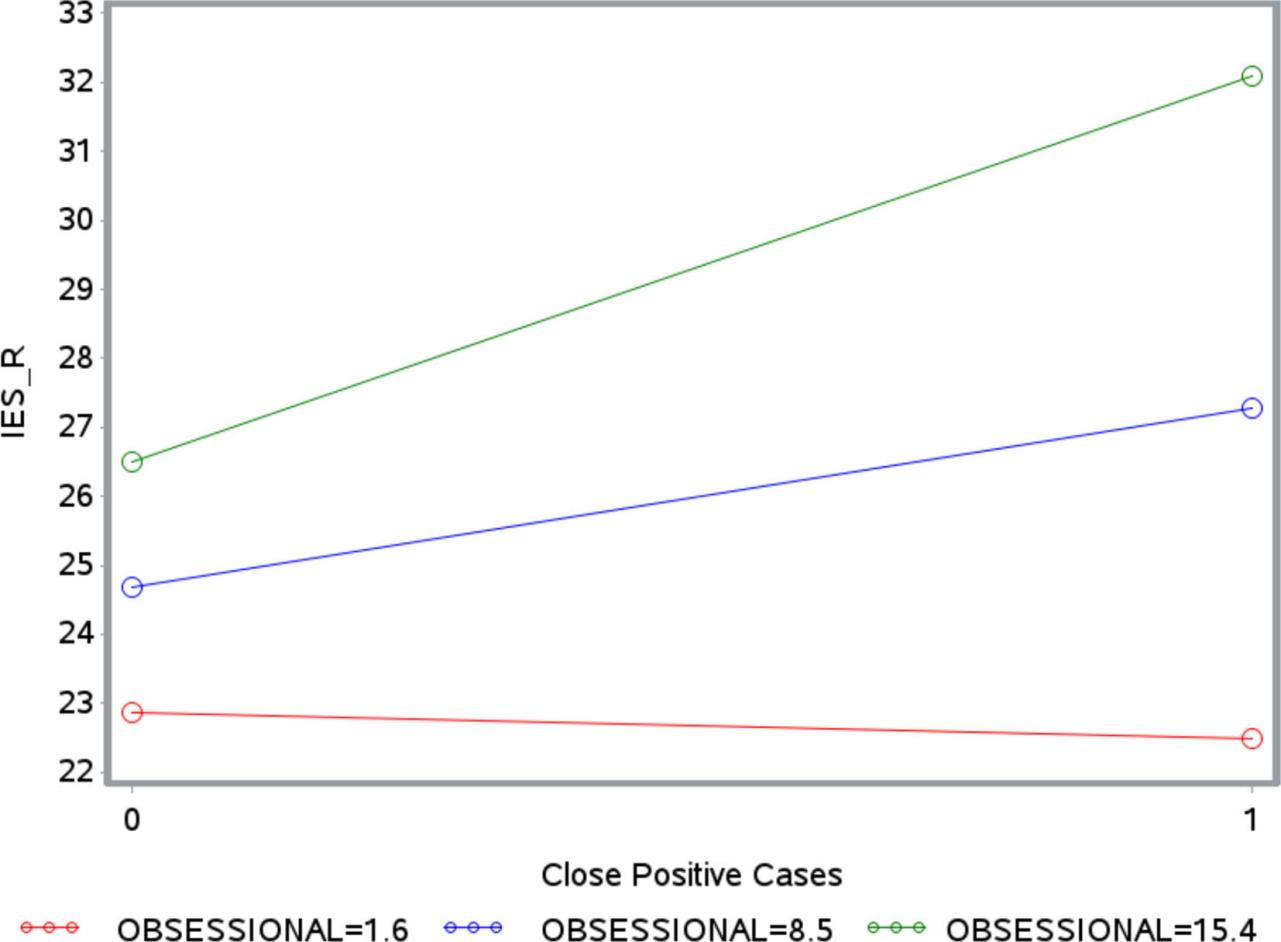
Moderating effect of obsessional defenses on the association between close positive cases and Impact of Event Scale-Revised (IES-R). The number 0 on the x-axis indicates the absence of close positive cases. The number 1 on the x-axis indicates the presence of close positive cases. The label OBSESSIONAL refers to defense mechanisms belonging to the obsessional defense level. The red line, blue line, and green line indicate values of obsessional defense level of 1.6, 8.06, and 15.4%, respectively.

There was a significant moderation effect only for obsessional defenses, for both GSI (β = 0.01, Δ*R^2^* = 0.0008, *p* = 0.026) and IES-R (β = 0.43, Δ*R^2^* = 0.0016, *p* = 0.002). As illustrated in **Figures 1**, **2**, the strength of the relationship between the number of positive coronavirus cases nearby and GSI or IES-R increases with the level of obsessional mechanisms. The Johnson-Neyman technique revealed that the relationship between positive coronavirus cases nearby and GSI was significant for all values of obsessional mechanisms above 8.06%, but not significant for values below 8.06%. The relationship between positive coronavirus cases nearby and IES-R was significant for levels of obsessional mechanisms above 6.71%.

## Discussion

The present study is timely in view of recent recommendations to assess effects on individual and population mental health during the COVID-19 pandemic (2). While the mean distress scores in this sample were not elevated, a sizable proportion still scored within clinical ranges for overall distress (35.6%), depression (37.8%), anxiety (51.1%), and PTSD (29.4%), indicating significant psychological distress across all domains assessed. Because of our sampling method we do not know if this generalizes to the whole Italian population, which includes people who do not use social media, but the significant proportion of this sample that was in distress makes understanding the correlates meaningful. Similarly, our findings revealed important associations between sociodemographic variables and risk factors for endorsing post-traumatic psychological distress during the COVID-19 pandemic and identified key implicit emotion regulation processes that might moderate this distress. Defenses on every level of the hierarchy were significantly associated with the report of PTSS, positively or negatively. Italians experienced varying levels of distress during the first week of lockdown, depending on their age, gender, lifestyle, traumatic experience related to the virus spread, and defensive functioning.

With regard to the first hypothesis, that some individuals would be at higher risk of psychological distress than others, results confirmed previous findings that younger age and female gender increased the risk of mental health problems (5). This is instructive with regard to COVID-19 given that older individuals are at greater risk of mortality. Interestingly, COVID-19 exposure was not a significant predictor of psychopathology, whereas having positive cases nearby, more days on lockdown, and having to relocate because of COVID-19 were all related to higher levels of distress.

According to research on the psychological impact of COVID-19 (4), we interpreted these findings to indicate that having symptoms was more associated with disruptions and a sense of threat related to the outbreak than living in a region highly hit by the COVID-19.

The second hypothesis, that implicit emotion regulation capacities (e.g., defense mechanisms) would be associated with the risk for PTSD, was fully confirmed. Higher levels of defensive functioning (ODF) were associated with lower levels of overall psychological distress, particularly depressive and anxiety symptoms, and PTSD, sharing respectively 25.60 and 11.63% of the variance. Specifically, the likelihood of having PTSD increased 71% for each unit decrease in ODF. Our findings are consistent with reports that the defense hierarchy is highly associated with measures of symptoms and functioning (41–43). Furthermore when defenses change, symptoms and functioning change in predictable ways (44, 45). As the current pandemic leads to more cases and greater time in lockdown, individual defensive functioning may slip and distress increase. This suggests the importance of considering the use of interventions that foster adaptive implicit emotion regulation (46–48).

Findings confirm that different defense levels are associated with varying levels of adaption to stressors and, hence, overall distress, depression, anxiety, and post-traumatic symptoms. Mature defenses generally accomplish this without distorting reality, while allowing the awareness of personal wishes, fears, and emotional responses, thereby optimizing adapting. Lower defense levels involve some tradeoffs that lead to degrees of poorer adaptation. In order to avoid conflictual motives or feelings, other neurotic defenses hide some important aspect of ideas, motives or feelings, trading off awareness of problems for nameless anxiety. Minor image-distorting defenses temporarily up-regulate self-esteem and sense of adequacy by distorting the images of others or oneself, but without effect on stressors. Disavowal defense deny, cover-up, or mis-attribute the sources of stress to avoid shame and responsibility. Major-image-distorting defenses distort reality into all good or bad images to avoid threats and powerlessness, but failing to see all sides of problems. Action defenses bypass inhibitions and express motives and emotions immediately without considering unpalatable consequences. Finally, the group of depressive defenses showed the strongest positive association to the measures of distress, specifically depression and anxiety, and of PTSD symptoms, consistent with other studies (44, 45).

Consistent with other research (49), moderation analyses found that higher levels of obsessional defenses were associated with increasing distress and trauma symptoms among individuals who know COVID-19 cases. Because obsessional defenses keep facts undistorted, they do not impair functioning; however, by minimizing affective experience, they lead to failures to address one’s emotional life and leave the individual vulnerable to distress, as found in other studies (35, 45, 50).

Finally, impaired handling of emotional responses to the COVID-19 disruptions and threats may be associated with the PTSD symptoms of avoidance and hyperarousal. While not addressed in this report, professional interventions with individuals who are avoiding charged feelings about the COVID-19 outbreak may mitigate the development or exacerbation of PTSD symptoms among those at risk. We also await the replication of our findings in other geographic areas and especially with longitudinal data to examine evidence that any improvements in defensive functioning among those at risk may be associated with diminished distress and trauma symptoms.

This survey used a snowball sampling method *via* social media contacts, which is not systematic but haphazard, and possibly biased in some ways. However, the large number of respondents reflected all Italian regions and a wide range of ages. As a cross-sectional study, causal relationships cannot be determined, nor the sequence in which factors operate. For instance, higher defensive functioning prior to the outbreak should protect individuals from developing symptoms once the outbreak occurred, but this study cannot establish temporality. It could also be that individuals who endorse symptoms during the outbreak tend to score lower on defensive functioning as an effect of self-report while distressed.

Despite its limitations, this study has relevant clinical implications. Findings indicate that defensive functioning, an operationalization of implicit emotion regulation, likely has an impact on the experience of pandemic-associated distress. These mechanisms appear to moderate the relationship between other risk factors—such as knowing someone with COVID-19—and symptoms of distress and PTSD. The key role of implicit emotion regulation in dealing with stressful life events is particularly important for vulnerable people, such as patients with chronic physical and mental illness (51–56).

## Conclusion

The world is experiencing one of the worst pandemics in recent history with daily exponential increases in diagnosis and mortality. Mental health professionals are observing the psychological impact of such a disaster and are trying to respond with adjusted interventions (57–60). In line with Brooks and colleagues (5), these findings suggest that psychological impact of COVID-19 and associated quarantining must be seriously considered by the healthcare system, with particular attention to those in high-risk categories. Automatic capacities for emotion regulation (e.g., defense mechanisms) may moderate the deleterious effects of pandemic-related trauma (61, 62). Mental health professions may need to screen for poorer defensive functioning in the face of high levels of subjective distress, especially depressive and anxiety symptoms, and PTSD symptoms to provide targeted interventions in order to alleviate distress (63–65).

## Data Availability Statement

The raw data supporting the conclusions of this article will be made available by the authors, without undue reservation.

## Ethics Statement

The studies involving human participants were reviewed and approved by Committee on Bioethics of the University of Pisa. The patients/participants provided written informed consent to participate in this study.

## Author Contributions

MG conceived the research study. MG, CC, and GO created the online survey. MG and JP developed and validated the novel DMRS-SR-30 measure. SZ-M performed data analyses. TP, SZ-M and MG wrote the first draft of the manuscript. JP contributed to the interpretation of the results and critically reviewed the final draft of the manuscript. All authors contributed to the article and approved the submitted version.

## Conflict of Interest

The authors declare that the research was conducted in the absence of any commercial or financial relationships that could be construed as a potential conflict of interest.
